# Supplementary material for: Vein Network and Climatic Factors Predict the Leaf Economic Spectrum of Desert Plants in Xinjiang, China
Source: Plants (Basel). 2023 Jan 28;12(3):581. doi: 10.3390/plants12030581 (PMC9920464; doi:10.3390/plants12030581)
Supplement: Supplementary file 1 [file plants-12-00581-s001.zip › plants-2171077-supplementary.pdf]

Table S1 Loadings of the principal components analyses (PCA) for the 10 leaf economic traits

Leaf traits	PC1 (63.1%)	PC1(20.2%)
N_m	-0.45225	0.805935
N_a	-0.96884	0.192567
P_m	0.457768	0.83517
P_a	-0.74719	0.538153
LMA	-0.95398	-0.1862
A_a	-0.71161	0.155754
A_m	0.903924	0.363774
Rd_a	0.65915	0.210466
Rd_m	0.905311	0.256626
PNUE	0.954325	-0.20521

LMA. leaf mass per area; A_a. carbon assimilation rates on area basis; A_m. carbon assimilation rates on mass basis; N_a. leaf nitrogen content on area basis; N_m. leaf nitrogen content on mass basis; P_a. leaf phosphorus content on area basis; P_m. leaf phosphorus content on mass basis; Rd_a. dark respiration rate on area basis; Rd_m. dark respiration rate on mass basis; *PNUE*. photosynthetic nitrogen use efficiency.

Table S2 Loadings of the principal components analyses (PCA) for the 10 leaf economic traits of three plant species

A. sparsifolia	Leaf traits	PC1 (82.8%)	PC2 (11.8%)
	N_m	-0.325	-0.213
	N_a	-0.346	-0.044
	P_m	-0.32	-0.045
	P_a	-0.345	0.075
	LMA	-0.335	0.155
	A_a	-0.331	-0.128
	A_m	0.318	-0.234
	Rd_a	-0.059	0.896
	Rd_m	0.327	0.215
	PNUE	0.347	-0.019
K. caspia	Leaf trait	PC1 (82.3%)	PC2 (10.1%)
	N_m	0.251	-0.228
	N_a	-0.335	-0.08
	P_m	0.345	-0.133
	P_a	-0.083	-0.949
	LMA	-0.348	0.027
	A_a	-0.321	0.098
	A_m	0.346	0.001
	Rd_a	0.343	-0.001
	Rd_m	0.348	-0.001
	PNUE	0.340	0.114
A. venetum	Leaf traits	PC1 (75.5%)	PC2 (16.2%)
	N_m	-0.28	-0.442
	N_a	-0.358	0.057
	P_m	-0.307	-0.314
	P_a	-0.335	0.303
	LMA	-0.324	0.359
	A_a	-0.294	0.181
	A_m	0.290	-0.399
	Rd_a	0.246	0.520
	Rd_m	0.355	0.030
	PNUE	0.354	0.138

Note: The percentages in parentheses are proportions of variance explained. *LMA*.

leaf mass per area; A_a. carbon assimilation rates on area basis; A_m. carbon assimilation rates on mass basis; N_a. leaf nitrogen content on area basis; N_m. leaf nitrogen content on mass basis; P_a. leaf phosphorus content on area basis; P_m. leaf phosphorus content on mass basis; Rd_a. dark respiration rate on area basis; Rd_m. dark respiration rate on mass basis; *PNUE*. photosynthetic nitrogen use efficiency.

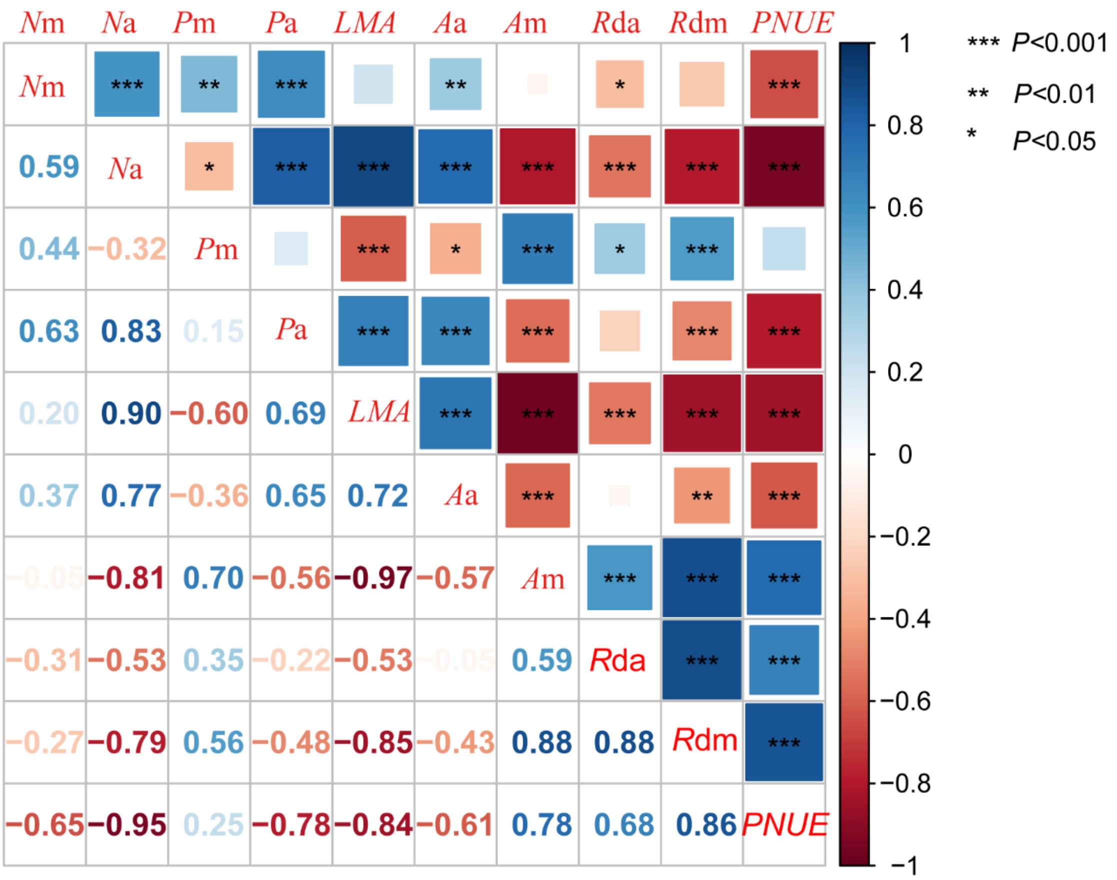

Figure S1 Pearson correlation between leaf economic traits. The correlation coefficient (r) was on the lower left. The size of the squares represents the strength of the correlation. ***. *P*<0.001; **. *P*<0.01; *. *P*<0.05. *LMA*. leaf mass per area; A_a. carbon assimilation rates on area basis; A_m. carbon assimilation rates on mass basis; N_a. leaf nitrogen content on area basis; N_m. leaf nitrogen content on mass basis; P_a. leaf phosphorus content on area basis; P_m. leaf phosphorus content on mass basis; Rd_a. dark respiration rate on area basis; Rd_m. dark respiration rate on mass basis; *PNUE*. photosynthetic nitrogen use efficiency.

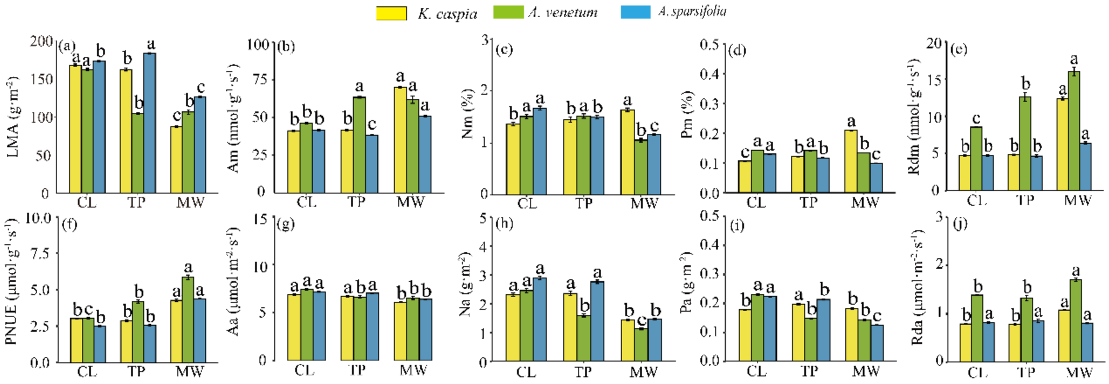

Fig. S2 Leaf economic trait differences of three desert plants. CL. Cele; MW. Mosuowan; TP. Turpan; *LMA*. leaf mass per area; A_{m}. leaf carbon assimilation rate on mass; N_{m}. leaf nitrogen concentration on mass; P_{m}. leaf phosphorus concentration on mass; Rd_{m}. leaf dark respiration rate on mass; *PNUE*. photosynthetic nitrogen use efficiency; A_{a}. leaf carbon assimilation rate on the area; N_a. leaf nitrogen concentration in the area; P_a. leaf phosphorus concentration on the area; Rd_a. leaf dark respiration rate in the area.

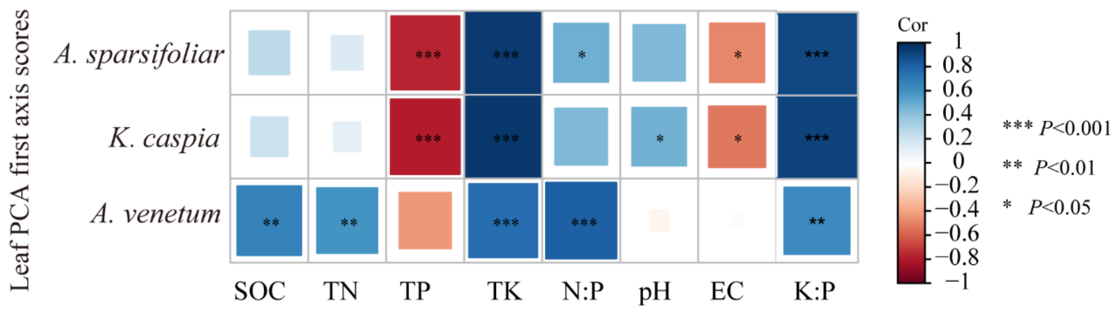

Figure S3 Pearson correlation between PCA first axis scores of leaf economic traits and soil properties. The correlation coefficient (r) was on the lower left. ***. *P*<0.001; **. *P*<0.01; *. *P*<0.05. SOC. Soil organic carbon; TN. total nitrogen; TP. total phosphorus; TK. total potassium; EC. electrical conductivity.

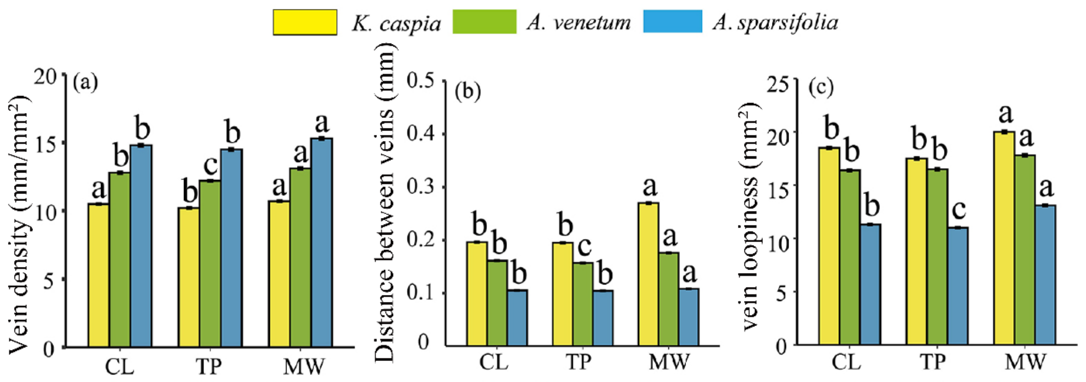

Figure S4 Leaf vein trait differences of three desert plants. CL.Cele; MW. Mosuowan; TP.

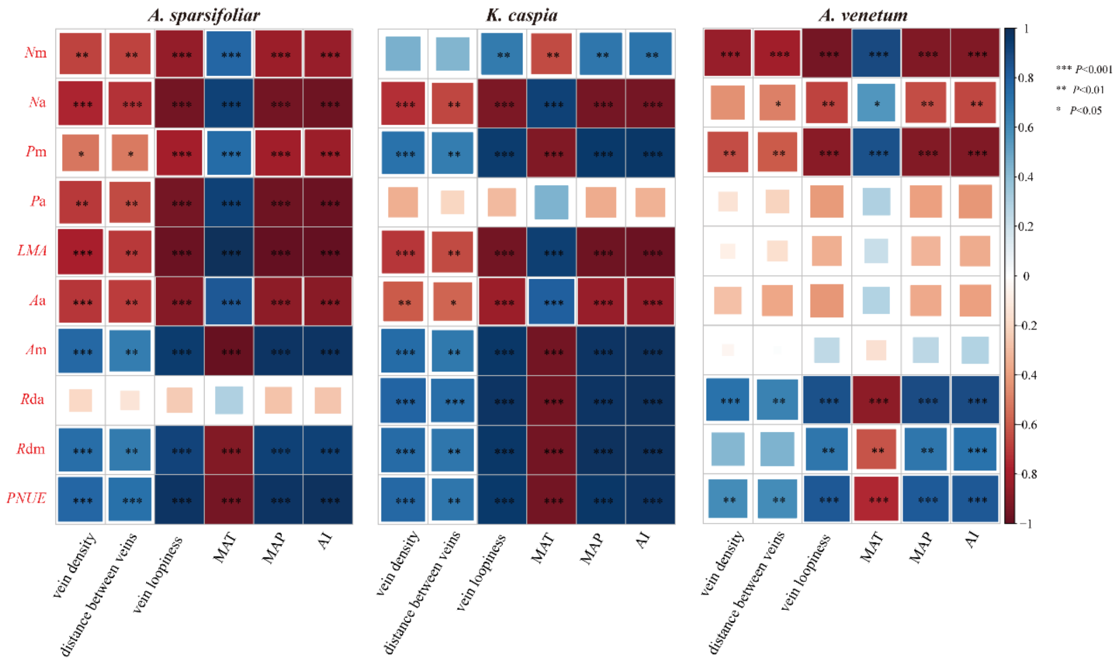

Figure S5 Correlation analysis of leaf traits, climate factors, and leaf vein characteristics. MAT. mean annual temperature; MAP. mean annual precipitation; AI. aridity index; *LMA*, leaf mass per area; A_m, leaf carbon assimilation rate on mass; N_m, leaf nitrogen concentration on mass; P_m, leaf phosphorus concentration on mass; Rd_m, leaf dark respiration rate on mass; *PNUE*, photosynthetic nitrogen use efficiency; A_a, leaf carbon assimilation rate on the area; N_a, leaf nitrogen concentration in the area; P_a, leaf phosphorus concentration on the area; Rd_a, leaf dark respiration rate in the area.
